# The effect of cooled endotracheal tubes on postoperative sore throat: a randomized controlled study

**DOI:** 10.3325/cmj.2025.66.256

**Published:** 2025-08

**Authors:** Sibel Çatalca, Özlem Özmete, Oya Yalçın Çok, Caner İncekaş, Nesrin Bozdoğan Özyılkan

**Affiliations:** 1 Department of Anesthesiology and Reanimation, Başkent University, Adana Dr. Turgut Noyan Hospital, Adana, Turkey; 2 Department of Anesthesiology and Reanimation, Penn State Health Milton S. Hershey Medical Center, Hershey, PA; 3 Department of Statistics, Başkent University, Ankara, Turkey

## Abstract

**Aim:**

To evaluate the effect of cooled endotracheal tubes on the incidence of postoperative sore throat, hoarseness, coughing, and dysphagia after endotracheal intubation.

**Methods:**

This randomized controlled trial enrolled patients undergoing elective surgery under general anesthesia with endotracheal intubation at Adana Dr Turgut Noyan Hospital between April and September 2023. The patients were randomly assigned to be intubated with endotracheal tubes refrigerated at +4 °C for 4-6 hours before intubation (n = 66) or with endotracheal tubes kept at the operating room temperature (+20-23 °C) (n = 70). The incidence of sore throat, hoarseness, coughing, and dysphagia was recorded at 1, 4, 12, and 24 hours postoperatively.

**Results:**

The groups did not significantly differ in the incidence of sore throat, hoarseness, and coughing at 1 hour and 24 hours. They also did not differ in the incidence of dysphagia at 24 hours.

**Conclusion:**

Cooled endotracheal tubes are not superior to tubes kept at operating room temperature in reducing postoperative sore throat, hoarseness, coughing, or dysphagia.

ClinicalTrials.gov identifier: NCT05834179

Sore throat is usually described as pain or difficulty swallowing, while some patients report a scratchy or irritated sensation (1). Postoperative sore throat (POST) is a common complication following endotracheal intubation (ETI) (up to 60%) and a frequent cause of patient dissatisfaction. It primarily results from trauma and inflammation of the airway and laryngeal mucosa caused by the laryngoscope or endotracheal tube (ETT) (2-4). The incidence of POST varies according to sex, smoking status, Mallampati score, ETT size, the number of intubation attempts, cuff pressure, use of nitrous oxide, patient positioning during surgery, and the duration of intubation, anesthesia, and surgery (2,4-8).

POST is pharmacologically treated with lidocaine, dexamethasone, dexmedetomidine, remifentanil, and benzydamine hydrochloride. However, some of these agents may produce systemic or local side effects, including nausea, vomiting, coughing, respiratory depression, and hemodynamic instability. Additionally, the effectiveness of these methods remains inconsistent or lacks robust evidence (2,9-14). Therefore, the use of various non-pharmacological approaches in reducing POST has also been investigated (15,16).

Cold application is a non-invasive, cost-effective, and simple method for pain management. It exerts its effects by increasing the pain threshold, inhibiting nocireceptors, causing vasoconstriction, and reducing the metabolic enzyme activity, muscle spasm, capillary permeability, local blood flow, edema, and inflammation (17,18). Cold application can decrease pain at the surgical site and lower postoperative analgesic requirements in various types of surgeries (17,19,20). Localized cooling has been shown to reduce muscarinic cholinoceptor-mediated inositol phosphate and inositol 1,4,5-trisphosphate accumulation, and basal phosphoinositidase C activity in tracheal mucosa, as well as to affect airway smooth muscle tone. It also inhibits calcium influx, contributing to the relaxation of airway smooth muscle (21,22). Therefore, different cold application techniques, such as oropharyngeal irrigation with cold saline, use of ice cubes, and consuming ice cream, have been used to reduce POST (23-25).

However, the effect of using a cooled ETT on POST, hoarseness, coughing, and dysphagia remains unknown. We hypothesized that a cooled ETT could reduce POST by minimizing airway irritation. Therefore, this study aimed to evaluate the effects of cooled endotracheal tubes on POST, hoarseness, coughing, and dysphagia after ETI.

## PATIENTS AND METHODS

### Study design

This single-center, randomized controlled trial enrolled patients aged 18-65 years with American Society of Anesthesiologists (ASA) Physical Status I or II and scheduled for elective surgery under general anesthesia with ETI at Adana Dr Turgut Noyan Hospital between April and September 2023. The study protocol was approved by the Baskent University Institutional Review Board. Written informed consent was obtained from all participants during the preoperative evaluation. All procedures were performed in accordance with the Declaration of Helsinki.

Non-inclusion criteria were refusal to participate; preexisting sore throat, hoarseness, coughing, or dysphagia; a history of gastroesophageal reflux, tracheostomy, tracheotomy, pregnancy, or anatomical abnormalities of the neck; a body mass index (BMI)<18 or ≥30 kg/m^2^; a history of malignant hyperthermia or psychiatric disorders; uncooperativeness; ETI within the previous 30 days; a Mallampati score of >2; procedures involving the thyroid, cervical spine, cranium, neck, or laparoscopic surgeries, as well as tonsillectomy or septorhinoplasty; and an expected surgery duration of ≥6 hours or <1 hour. Exclusion criteria were inability to mask ventilate, the use of a videolaryngoscope, or >2 attempts for successful intubation; remifentanil infusion; the use of dexamethasone, sugammadex, and/or a nasogastric tube; and postoperative mechanical ventilation or reintubation.

### Randomization and allocation

Patients were randomly assigned to be intubated with either a cooled ETT or an operating room (OR)-temperature ETT using a randomization website (www.randomizer.org). Randomization was conducted via a computer-generated random-number list. An anesthesia technician unrelated to the study created opaque-sealed and sequentially numbered envelopes. After anesthesia induction, an anesthesiologist who was blinded to the group allocation opened the envelopes. All patients and anesthesiologists, except those responsible for measuring the ETT temperature and intubation, were blinded to group assignment. All postoperative evaluations were conducted by an independent anesthesiologist who was blinded to all perioperative procedures.

### Endotracheal tube preparation

An anesthesia technician uninvolved in the allocation procedure prepared a high-volume, low-pressure cuffed polyvinyl chloride ETT (Chilecom, Chilecom Medical Devices Co. Ltd, Boluo, China). The inner diameter of the ETT was 7.0 mm for women and 8.0 mm for men. The ETTs prepared for the intervention group were stored in a refrigerator at +4 °C for 4-6 hours before intubation. Those prepared for the control group were kept at the operating room temperature (+20-23 °C). In both groups, the cuff was fully deflated, and the ETT temperature was measured at the cuff level immediately before intubation using a non-contact infrared forehead thermometer (PlusMED, Shenzhen Everbest Machinery Industry Co. Ltd, Shenzhen, China).

### Anesthesia

A standardized general anesthesia protocol was applied to all patients. Continuous monitoring included electrocardiography, pulse oximetry, and noninvasive blood pressure measurement. Anesthesia was induced with propofol (2 mg/kg), fentanyl (1 μg/kg), and rocuronium (0.6 mg/kg). An oropharyngeal airway was inserted in case of difficulty with face mask ventilation. After two minutes of controlled mask ventilation, direct laryngoscopy was performed using a Macintosh blade (size 3 for women and size 4 for men). All intubations were performed by an anesthesiologist with more than 10 years of clinical experience. ETT cuffs were inflated with dry air to a pressure of 20-30 cm H_2_O using a manometer (VBM Medizintechnik GmbH, Sulz, Germany). No intubation stylet was routinely used. The accuracy of intubation was determined using a capnograph. Anesthesia was maintained with sevoflurane (2%-3%) in an oxygen/nitrous oxide mixture (2:2 L/min). Volume-controlled ventilation with a tidal volume of 8 mL/kg and an adequate respiratory rate to keep the end-tidal carbon dioxide concentration between 30 and 40 mm Hg were used. A standardized extubation procedure was followed. Oropharyngeal secretions were gently suctioned, and neuromuscular blockade was reversed with neostigmine (0.04 mg/kg) and atropine (0.02 mg/kg). Patients were extubated when they fully recovered and were able to follow verbal commands.

### Data collection

Preoperatively, we recorded data on age, sex, BMI, smoking status, ASA score, and dental conditions. After anesthesia induction, the difficulty of mask ventilation was assessed using a three-point scale: 1) ventilation without an oral airway; 2) ventilation with an oral airway; 3) difficult mask ventilation (inadequate, unstable, or requiring two practitioners). The anesthesiologist performing laryngoscopy assessed each patient’s Cormack-Lehane score and the need for the back-up-rightward pressure maneuver. The resistance encountered during ETT passage through the vocal cords was graded on a four-point scale as follows: 0) none; 1) mild; 2) moderate; 3) severe. The temperature of the ETT cuff, the number of intubation attempts, intubation time (duration between the time of insertion of the laryngoscopic blade into the mouth and inflating the cuff), anesthesia time (duration from laryngoscopy to tracheal extubation), operation time (duration from surgical incision to the last suture), and the total intraoperative doses of fentanyl and rocuronium were recorded. During extubation, the presence of the following conditions was noted: 1) blood in the oral cavity through aspiration or at the tip of the ETT; 2) excessive secretions requiring extensive suctioning; 3) desaturation (SpO_2_≤93); 4) laryngospasm; and 5) coughing ≥2 times. Patients were transferred to the post-anesthesia care unit once they had fully recovered and were subsequently moved to the ward when their modified Aldrete score was >9.

Postoperative sore throat, hoarseness, and coughing were assessed at 1, 4, 12, and 24 hours following extubation. Dysphagia was evaluated at 12 and 24 hours. These outcomes were evaluated based on patient self-reports. Additionally, oropharyngeal injuries, such as hyperemia, petechiae, or hematoma, were examined using a tongue depressor and a light source at 1 and 24 hours.

The primary outcome of the study was the incidence of POST at 1 hour after surgery. The secondary outcomes were the incidence of hoarseness and coughing at 1, 4, 12, and 24 hours, and the incidence of dysphagia at 12 and 24 hours.

### Statistical analysis

The incidence of POST was previously reported to be 54% (15). For the present study, the required sample size was calculated to be 58 patients per group, assuming an alpha level of 0.05, a power of 0.80, and an effect size of 0.54. To account for a potential dropout rate of 20%, 140 patients were included. A 20% reduction in the incidence of POST with the use of a cooled ETT was considered clinically significant.

The Shapiro-Wilk test was used to assess the normality of data distribution. Continuous data are presented as medians with ranges, while categorical variables are expressed as frequencies and percentages. The differences between the groups were assessed with a Mann-Whitney U test or a Fisher-Freeman-Halton exact test. Dunn’s Bonferroni test was applied for *post-hoc* comparisons. A *P* value of <0.05 was considered significant. Statistical analysis was performed with SPSS, version 25.0 (IBM Corp., Armonk, NY, USA).

## RESULTS

**Figure 1:**
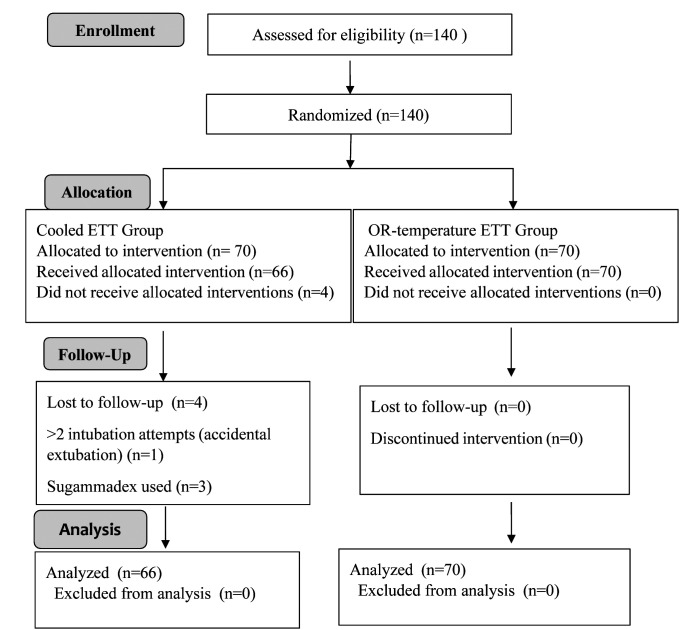
Study flowchart.

Initially, 140 patients were randomized into two groups. Four patients from the cooled ETT group were excluded from the study – one as a result of more than two intubation attempts (accidental extubation) and three as a result of sugammadex administration during extubation. The final analysis included data from 136 patients (Figure 1).

The two groups were comparable in terms of baseline characteristics, including demographic data, surgical details, and anesthesia parameters (Table 1). ETT temperature was 12.2 °C (10.9-13.5 °C) in the cooled ETT group and 20 °C (18.4-23 °C) in the control group (*P* < 0.001). There were no significant differences between the groups in the consumption of intraoperative rocuronium (50 mg [40-100] in both groups, *P* = 0.771) or fentanyl (125 μg [50-225] in both groups, *P* = 0.769).

**Table 1:** Demographic characteristics, anesthesia-related parameters, and surgical parameters in the cooled ETT and OR-temperature ETT groups*^†^

	Cooled ETT (n = 66)	OR-temperature ETT (n = 70)	p
Age	48.5 (19-65)	48 (18-65)	0.742
Sex			0.978
female	47 (71.2)	50 (71.4)	
male	19 (28.7)	20 (28.5)	
Body mass index	27.1 (18.7-29.8)	27.1 (20.3-29.9)	0.905
Edentulism	5 (7.5)	6 (8.5)	0.831
Smoking history	18 (27.2)	21 (30)	0.725
ASA score (1/2)	34 (51.5)/ 32 (48.4)	29 (41.4)/ 41 (58.5)	0.238
Mallampati score			0.408
1	14 (21.2)	11 (15.7)	
2	52 (78.7)	59 (84.2)	
**Type of surgery**			
breast	29 (43.9)	35 (50)	
abdominal	29 (43.9)	25 (35.7)	0.797
urological	6 (9)	7 (10)	
orthopedic	2 (3)	3 (4.2)	
Mask ventilation score			0.973
1	51 (77.2)	53 (75.7)	
2	14 (21.2)	16 (22.8)	
3	1 (1.5)	1 (1.4)	
Cormack Lehane score			0.302
1	36 (54.5)	38 (54.2)	
2a	18 (27.2)/	25 (35.7)	
2b	12 (18.1)	7 (10)	
The resistance encountered during ETT passage			0.232
0	53 (80.3)	53 (75.7)	
1	13 (19.7)	14 (20)	
2	0	3 (4.2)	
3	0	0	
Need for the BURP maneuver	12 (18.1)	8 (11.4)	0.266
Number of attempts			0.713
1	62 (93.9)	67 (95.7)	
2	4 (6)	3 (4.3)	
The duration of intubation (seconds)	20 (10-26)	20 (10-35)	0.956
The duration of surgery (min)	95 (60-330)	101 (60-328)	0.562
The duration of anesthesia (min)	113.5 (65-345)	120 (70-355)	0.565

*Abbreviations: OR – operating room; ASA – American Society of Anesthesiologists; CL – Cormack Lehane; ETT – endotracheal tube; BURP – back, up, rightward, pressure.

†Data are expressed as the median (min-max) or number (%).

The groups did not significantly differ in terms of adverse events during extubation (Table 2), the number of patients receiving analgesics during the intraoperative and postoperative periods (Table 3), and oropharyngeal injury at 1 and 24 hours postoperatively (Table 4). No oropharyngeal hematoma was detected in either group.

**Table 2:** Adverse events during extubation in the cooled ETT and OR-temperature ETT groups*

	Cooled ETT (n = 66)	OR-temperature ETT (n = 70)	p
	n (%)	n (%)	
Blood in the oral cavity through aspiration	4 (6)	6 (8.5)	0.746
Blood at the tip of the ETT	2 (3)	3(4.2)	1.000
Excessive secretion requiring intensive aspiration	11 (16.6)	6 (8.5)	0.154
Desaturation	2 (3)	2 (2.8)	1.000
Coughing ≥2 times	3 (4.5)	2 (2.8)	0.674
Laryngospasm	4 (6)	4 (5.7)	1.000

*Abbreviations: ETT – endotracheal tube; OR – operating room.

**Table 3:** İntraoperative and postoperative analgesic agents consumption in the cooled ETT and OR-temperature ETT groups*

Analgesic agent	Cooled ETT (n = 66)	OR-temperature ETT (n = 70)	p
	n (%)	n (%)	
Intraoperative			
tenoxicam	57 (86.3)	64 (91.4)	0.346
paracetamol	54 (81.8)	57 (81.4)	0.953
tramadol	40 (60.6)	40 (57.1)	0.682
morphine sulfate	25 (37.8)	29 (41.4)	0.672
Postoperative			
paracetamol	52 (78.7)	58 (82.8)	0.546
tenoxicam	10 (15.1)	14 (20)	0.459
tramadol	40 (60.6)	44 (62.8)	0.787
pethidine	4 (6)	1 (1.4)	0.199

*Abbreviations: ETT – endotracheal tube; OR – operating room.

**Table 4:** Oropharyngeal injury in the cooled ETT and OR-temperature ETT groups at 1 hour and 24 hours postoperatively*

	Cooled ETT (n = 66)	OR-temperature ETT (n = 70)	p
	n (%)	n (%)	
1 h			
hyperemia	35 (53)	41 (58.5)	0.515
petechia	1 (1.5)	4 (5.7)	0.367
24 h			
hyperemia	12 (18.1)	12 (17.1)	0.874
petechia	─	2 (2.8)	0.497

*Abbreviations: ETT – endotracheal tube; OR – operating room.

At 1, 4, 12, and 24 hours after surgery, the groups did not significantly differ in the incidences of POST, hoarseness, coughing, and dysphagia (Table 5). In subgroup analysis, the incidence of POST at 1 hour after surgery was significantly higher in women than in men (n = 52, 53.6% vs n = 13, 33.3%; *P* = 0.032). Additionally, patients who experienced POST at 1 hour had a significantly longer duration of anesthesia (140 minutes [70-355] vs 94 minutes [65-327]; *P* < 0.001).

**Table 5:** The incidence of postoperative sore throat, hoarseness, coughing, and dysphagia in the cooled ETT and OR-temperature ETT groups at 1, 4, 12, and 24 hours postoperatively*

		Cooled ETT (n = 66)	OR-temperature ETT (n = 70)	p
		n (%)	n (%)	
1 h				
POST	no	38 (57.58)	33 (47.14)	0.223
	yes	28 (42.42)	37 (52.86)	
hoarseness	no	38 (57.58)	41 (58.57)	0.906
	yes	28 (42.42)	29 (41.43)	
coughing	no	60 (90.91)	67 (95.71)	0.315
	yes	6 (9.09)	3 (4.29)	
4 h				
POST	no	37 (56.06)	37 (52.86)	0.708
	yes	29 (43.94)	33 (47.14)	
hoarseness	no	48 (72.73)	45 (64.29)	0.290
	yes	18 (27.27)	25 (35.71)	
coughing	no	60 (90.91)	66 (94.29)	0.523
	yes	6 (9.09)	4 (5.71)	
12 h				
POST	no	45 (68.18)	51 (72.86)	0.550
	yes	21 (31.82)	19 (27.14)	
hoarseness	no	54 (81.82)	58 (82.86)	0.874
	yes	12 (18.18)	12 (17.14)	
coughing	no	61 (92.42)	68 (97.14)	0.264
	yes	5 (7.58)	2 (2.86)	
dysphagia	no	62 (93.94)	65 (92.86)	1.000
	yes	4 (6.06)	5 (7.14)	
24 h				
POST	no	53 (80.30)	61 (87.14)	0.279
	yes	13 (19.70)	9 (12.86)	
hoarseness	no	60 (90.91)	64 (91.43)	0.915
	yes	6 (9.09)	6 (8.57)	
coughing	no	65 (98.48)	70 (100)	0.485
	yes	1 (1.52)	─	
dysphagia	no	63 (95.45)	66 (94.29)	1.000
	yes	3 (4.55)	4 (5.71)	

*Abbreviations: ETT – endotracheal tube; OR – operating room; POST – postoperative sore throat.

## DISCUSSION

In this study, the use of a cooled ETT did not significantly reduce the incidence of POST compared with an ETT at OR temperature. Furthermore, the incidences of hoarseness, coughing, dysphagia, and oropharyngeal injury were comparable between the groups.

To maintain a standardized protocol, all ETT cuffs were inflated with air, and all patients were operated on in the supine position. Previous studies reported a significant reduction in POST incidence when using a size 6.5 ETT instead of a size 7.0 in women, and a size 7.5 instead of an 8.0 in men (6). However, since smaller ETT sizes are associated with intraluminal obstruction, kinking of ETT, increased airway resistance, and aspiration (26), we preferred a 7-sized ETT for women and an 8-sized ETT for men.

Due to negative or conflicting results associated with pharmacological treatments, studies evaluated various non-pharmacological methods for POST reduction, such as intubation using ETTs with thermal softening or lubrication with normal saline (NS) (15,16). NS can reduce the friction between the airway mucosa and the ETT. However, ETTs lubricated with NS have not been shown to reduce POST, likely because NS lacks anti-inflammatory effects, and POST is caused by ETT-induced inflammation. Furthermore, this method may also lead to ETT contamination (16). Similarly, thermally softening ETTs in an NS bottle at +40°C could cause an infectious process or mucosal injury in the airway.

Cold therapy has been shown to reduce inflammation and neural signal transmission through vasoconstriction, suppression of metabolic activity, and inhibition of pro-inflammatory cytokines such as interleukin 6, interleukin 1β, nuclear factor kappa B, and prostaglandin E2 (17,27), although the exact mechanisms behind these processes are not fully understood. The anti-inflammatory properties and theoretical benefits of cold application on tracheal mucosa have prompted several research groups to explore various cooling methods during surgery (eg, cooling the tonsillar fossa and pharyngeal mucosa with cold water) and in the postoperative period (eg, ice cube absorption, cold vapor inhalation, ice cream consumption) (23-25). While some of these studies have reported positive effects (23-25), others have not found any significant benefit (28,29). Differences between our findings and those of studies with positive results may be explained in several ways. First, the method of cold application may play a critical role. Other studies employed pharyngeal cooling or postoperative cold vapor application, whereas our study evaluated the use of a cooled ETT during intubation. To the best of our knowledge, no prior studies investigated the effect of cooled ETTs on POST, hoarseness, coughing, and dysphagia following ETI. Another explanation may be related to the type of surgery performed. In previous studies, patients who underwent head and neck surgeries, such as thyroidectomy or tonsillectomy, may have been unable to distinguish between discomfort caused by surgical trauma and that caused by ETT-related inflammation. As a result, POST may have been underreported or misattributed (29,30). To address this issue, we excluded patients undergoing these types of surgeries. Another possible reason may be the application time. In our study, cooled ETT was applied during intubation due to the nature of intubation process. According to a recent meta-analysis, pre-intubation non-pharmacological interventions significantly reduced the incidence of POST, while interventions applied during intubation did not (31). Pre-intubation interventions act proactively, creating a protective barrier before mucosal injury occurs. In contrast, interventions during or after intubation are reactive, attempting to mitigate inflammation after tissue damage has already occurred, and thus have limited effectiveness. The authors of the meta-analysis also emphasized the importance of incorporating pre-intubation non-pharmacological strategies into clinical practice and enhanced recovery after surgery protocols to improve patient outcomes. The meta-analysis covered both cold and non-cold applications, such as gum chewing, thermal softening of ETTs with warm saline, and gargling. Therefore, more research is needed to determine the specific role and optimal timing of cold applications in preventing ETT-related complications.

Our study suffers from some limitations. First, the single-center setting may limit the generalizability of the findings. Second, due to the nature of the study, the anesthesiologists who measured the ETT temperatures and performed the intubations were aware of group assignments. Although blinding was not feasible during intubation, all postoperative evaluations were conducted by an independent anesthesiologist who was blinded to perioperative procedures. Third, as routine neuromuscular monitoring is not implemented in our institution, extubation was performed based on clinical assessments. Additionally, the perioperative analgesic regimen was not randomized between groups due to adherence to the hospital’s standard analgesia protocol.

In conclusion, cooled ETTs in patients undergoing general anesthesia were not superior to room-temperature ETTs in reducing the incidence of POST. Additionally, this method did not decrease the incidence of hoarseness, coughing, or dysphagia in the postoperative period. Therefore, future research should explore other non-pharmacological interventions that may more effectively reduce the incidence of POST and other complications following ETI.
